# Bench-to-bedside review: the effects of hyperoxia during critical illness

**DOI:** 10.1186/s13054-015-0996-4

**Published:** 2015-08-17

**Authors:** Hendrik J. F. Helmerhorst, Marcus J. Schultz, Peter H. J. van der Voort, Evert de Jonge, David J. van Westerloo

**Affiliations:** Department of Intensive Care Medicine, Leiden University Medical Center, Albinusdreef 2, Leiden, 2300 RC The Netherlands; Laboratory of Experimental Intensive Care and Anesthesiology, Academic Medical Center, Meibergdreef 9, Amsterdam, 1105 AZ The Netherlands; Department of Intensive Care Medicine, Academic Medical Center, Meibergdreef 9, Amsterdam, 1105 AZ The Netherlands; Department of Intensive Care Medicine, Onze Lieve Vrouwe Gasthuis, Oosterpark 9, Amsterdam, 1091 AZ The Netherlands; TIAS School for Business and Society, Tilburg University, Warandelaan 2, Tilburg, 5000 LE The Netherlands

## Abstract

Oxygen administration is uniformly used in emergency and intensive care medicine and has life-saving potential in critical conditions. However, excessive oxygenation also has deleterious properties in various pathophysiological processes and consequently both clinical and translational studies investigating hyperoxia during critical illness have gained increasing interest. Reactive oxygen species are notorious by-products of hyperoxia and play a pivotal role in cell signaling pathways. The effects are diverse, but when the homeostatic balance is disturbed, reactive oxygen species typically conserve a vicious cycle of tissue injury, characterized by cell damage, cell death, and inflammation. The most prominent symptoms in the abundantly exposed lungs include tracheobronchitis, pulmonary edema, and respiratory failure. In addition, absorptive atelectasis results as a physiological phenomenon with increasing levels of inspiratory oxygen. Hyperoxia-induced vasoconstriction can be beneficial during vasodilatory shock, but hemodynamic changes may also impose risk when organ perfusion is impaired. In this context, oxygen may be recognized as a multifaceted agent, a modifiable risk factor, and a feasible target for intervention. Although most clinical outcomes are still under extensive investigation, careful titration of oxygen supply is warranted in order to secure adequate tissue oxygenation while preventing hyperoxic harm.

## Introduction

Oxygen is a vital element in human survival and plays a major role in a diverse range of biological and physiological processes. In medical practice, it is among the most universally used agents for the treatment of critical illness [1] and part of the routine treatment in acute shock and emergency medicine [2]. To ensure sufficient oxygenation, oxygen therapy during mechanical ventilation, anesthesia, and resuscitation usually exceeds physiological levels. However, Renaissance physician Paracelsus noted: “nothing is without poison—the poison is in the dose”. This accounts for many aspects in medicine but may also be applicable to the oxygen molecule [3]. The concept of oxygen toxicity was described in the late 19th century following the pioneering efforts of James Lorrain Smith and Paul Bert, but it was not until a century later that the effects of hyperoxia were increasingly studied. Although several lines of evidence indicate that hyperoxia may be harmful, robust interventional studies are still limited. To develop adequate recommendations for optimal oxygen levels, it is important to extend our current understandings of hyperoxia-induced injury. The aim of this review is to provide a comprehensive overview of the effects of hyperoxia from the bench and the bedside. The first part will focus on established insights and recent experimental and translational advances; the latter part addresses pathophysiological concepts, clinical studies, and implications for therapy.

## Pathogenesis from the benchside

### Reactive oxygen species

Reactive oxygen species (ROS) are versatile molecules that can be essential in the regulation of intracellular signaling pathways and in host defense [4]. However, ROS have also repeatedly been postulated to be of major significance in tissue damage, organ dysfunction, and clinical disease. In regard to oxygen toxicity, it is frequently assumed that it is not oxygen itself that exerts toxic effects but merely the ROS that are generated as an undesirable by-product of adenosine triphosphate synthesis during aerobic cellular metabolism. The implications for the lungs are probably the most prominent as lung tissue is continuously and abundantly exposed to oxygen and its by-products. In physiological circumstances, ROS are formed in the electron transport chain during proton transport across the inner mitochondrial membrane. Mitochondrial oxidative phosphorylation is the most important source of oxygen species, but ROS may also be generated in response to exogenous stimuli, such as microbes, cytokines, and xenobiotics [5]. Antioxidant tasks are accomplished by enzymes as catalases, glutathione peroxidases, thioredoxins, and peroxyredoxins. These enzymes use electron donors in order to avoid the intermediate formation of the hydroxyl radical (OH∙), which is a strongly reactive oxidant. In this process, superoxide dismutase is an important antioxidant enzyme as it efficiently reduces the concentration of the superoxide anion (O_2_∙^–^) by facilitating its rapid conversion in hydrogen peroxide (H_2_O_2_) or oxygen (O_2_). In general, ROS generation from mitochondria increases with oxygen tension and is dependent on the clinical balance between the underlying condition and oxygen supply [6]. In response to bacterial invasion, neutrophils can also produce large amounts of ROS that may initially be beneficial in the host defense against several pathogens. Fortunately, the lungs are principally well protected against oxygen toxicity by adequate intra—and extracellular antioxidant activity. Besides this physiological activity, additional antioxidants can be recruited in the epithelial lining fluid [7]. However, when the production of ROS exceeds the limits of counteraction by antioxidant responses, ROS concentrations reach inadequate levels and a cellular state of oxidative stress manifests. Oxidative stress refers to the imbalance caused by increased ROS formation or deficient oxidant suppressors [8]. When antioxidant systems are insufficient during critical illness and mechanical ventilation, supplemental oxygen can cause accumulation of oxygen radicals and may initiate or perpetuate oxygen toxicity. Moreover, ROS control can be markedly influenced by aging, genetic factors, and pharmacochemical agents [6].

### Cell death

When the delicate homeostatic balance is disturbed, oxidative stress leads to damage of nucleic acids, proteins, and lipids, resulting in cell death by both apoptotic and necrotic pathways [9]. Necrosis is characterized by incomplete apoptosis and supported by integrity loss of the cell membrane and cytoplasmic swelling. Programmed cell death by apoptosis can be achieved through extrinsic or intrinsic pathways, concomitantly. The *extrinsic* pathway is triggered by extracellular signals that stimulate intracellular apoptotic cascades after binding the cell membrane. The *intrinsic* apoptotic pathway is initiated by increased mitochondrial ROS formation. Subsequently, the opening of transition pores is facilitated, making the outer mitochondrial membrane more permeable for pro-apoptotic components. These components can then pass to the cytoplasm and induce a state of intracellular stress. When this occurs in both endothelial and epithelial cells, lytic damage and cell death contribute to interstitial pulmonary edema and impaired gas exchange by means of alveolar collapse and disintegration of the alveolar-capillary barrier.

### Cell damage and inflammatory pathways

**Fig. 1:**
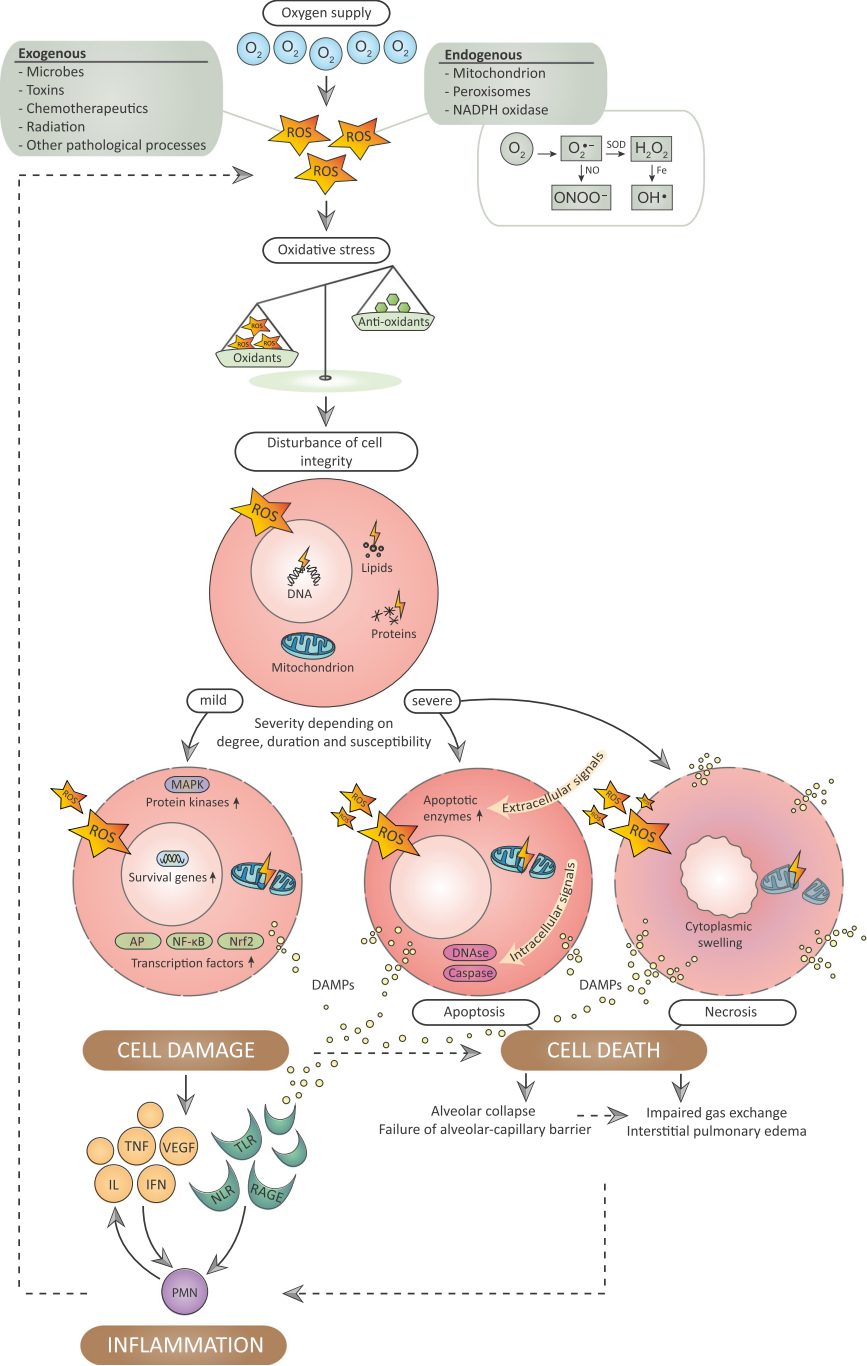
Vicious cycle of hyperoxia-induced cell injury. *AP* activator protein, *DAMP* damage-associated molecular pattern molecules, *H*_*2*_ *O*_*2*_ hydrogen peroxide, *IFN* interferon gamma, *IL* interleukin, *MAPK* mitogen-activated protein kinase, *NADPH* nicotinamide adenine dinucleotide phosphate, *NF-κB* nuclear factor kappa B, *NLR* nod-like receptor, *Nrf2* nuclear factor-2 erythroid related factor-2, *O*_*2*_ oxygen, *O*_*2*_^*·−*^superoxide, *OH* ^*·*^ hydroxyl radical, *ONOO* ^*−*^ peroxynitrite, *PMN* polymorphonuclear neutrophil, *RAGE* receptor for advanced glycation end products, *ROS* reactive oxygen species, *TLR* Toll-like receptor, *TNF* tumor necrosis factor, *VEGF* vascular endothelial growth factor

In addition to direct cell death by necrosis or apoptosis, cellular disruption caused by hyperoxia and ROS has been shown to release endogenous damage-associated molecular pattern molecules (DAMPs) that alert the innate immune system [10–12]. DAMPs, or alarmins, are cell fragments released during cellular dysfunction and sterile injury and act as pleiotropic modulators of inflammation. During oxidative stress, mitochondrial damage is a pivotal cause of extracellular hazardous content including both free radicals and DAMPs. Because they resemble bacterial DNA, circulating mitochondrial DAMPs are efficiently recognized by pattern recognition receptors and activate polymorphonuclear neutrophils (PMNs). Subsequently, PMNs release interleukins and contribute to a sterile inflammatory reaction and, ultimately, neutrophil-mediated organ injury. In response to hyperoxia-mediated ROS production, resident lung cells initiate the release of various cytokines. Chemotactic factors orchestrate the inflammatory response by attracting inflammatory cells to the pulmonary compartment. Recruited neutrophils and monocytes, in turn, are significant sources of additional ROS, conserving a vicious cycle leading to further tissue damage (Fig. 1). Under enduring conditions of injury to pulmonary epithelium and increasing alveolar permeability, cytokines can translocate from the alveolar space to the systemic circulation, creating a systemic inflammatory response, in which cytokines are efficiently activated and phagocytosis by alveolar macrophages is hampered [13]. Cytokine concentrations decrease after long-term exposure, suggesting that a fast upregulation of inflammatory action is followed by a gradual impairment of the innate immune system [14]. Besides mitochondrial damage, the inflammatory actions of oxygen are importantly modulated by hypoxia-inducible factor (HIF) [15, 16]. HIF-1α is thought to be upregulated during relative changes in oxygenation and accordingly responds to normoxia as a relative hypoxic state directly after hyperoxia. Through this mechanism, intermittent hyperoxia may trigger a paradoxical phenomenon in which the genetic expression of inflammatory mediators and erythropoietin is stimulated in the absence of true tissue hypoxia [17].

### Animal studies

Principal insights in hyperoxia-induced mechanisms have been obtained from experimental models. The first animal studies documented structural morphologic and biochemical changes in the lungs of a wide variety of animal species that were exposed to hyperoxia [18]. Pioneering studies using conscious dogs postulated that normobaric hyperoxia decreased metabolic rate and altered hemodynamics [19, 20]. These findings were reproduced in primates in which progressive pulmonary injury, interstitial edema, and inflammatory activation were observed [21]. In later experiments, biochemical effects of ROS and interventional targets on the molecular level were more intensively studied in spontaneously breathing animals in hyperoxic environments and showed both detrimental and protective potential [22–26]. Recent experiments were performed in mechanically ventilated rodents, rabbits, and pigs, mimicking the clinical environment of critically ill patients [27–31]. In this context, the interaction between injurious ventilation and concurrent hyperoxia was shown to transcend lung injury by alveolar distention alone [22, 32–35]. However, studies in mechanically ventilated animals are usually restricted to short exposure periods [32, 34–38], even though hyperoxia may induce time-dependent inflammation [23]. To improve our understanding of the impact of long-term exposure to both mechanical ventilation and hyperoxia, future studies involving mechanical ventilation of longer duration and with clinically relevant settings are essential for a robust representation of the ICU environment.

## Pathogenesis from the bedside

### Hyperoxia-induced tissue injury

Under normobaric circumstances, the side effects of oxygen are initially restricted to the lungs. However, when hyperoxia manifests for prolonged periods or under hyperbaric conditions, other organs are concurrently at risk as more oxygen is dissolved in plasma [6]. The amount of dissolved oxygen will readily increase at partial pressures of arterial oxygen (PaO_2_) exceeding 100 mm Hg. Oxyhemoglobin saturation is nearly complete when PaO_2_ approaches this level and the carrying capacity of hemoglobin is therefore quickly overcharged with increasing fractions of inspired oxygen (FiO_2_).

The harmful effects depend on underlying conditions, duration, and degree of the hyperoxic exposure. Rigid thresholds where harm exceeds the perceived benefits are not exactly known and may vary between subgroups [39]. Most pathophysiological changes originate rapidly and are rather universal effects, but the effects of hyperoxia are assumed to be time—and dose-dependent [40]. In general, excessive oxygen supply causes absorptive atelectasis by displacement of alveolar nitrogen. The progressive washout of nitrogen coincides with the abundant presence of oxygen in the alveoli which, driven by a steep pressure gradient, rapidly diffuses into the mixed venous blood. As a result, the alveolar volume is markedly reduced and leads to increased ventilation/perfusion mismatch by (partial) alveolar collapse and impaired gas exchange, which can be attenuated by applying positive end-expiratory pressure [41]. Impaired mucociliary clearance by hyperoxia contributes to obstructive atelectasis, and altered surfactant metabolism facilitates adhesive atelectasis through alveolar instability and collapse. Several lines of evidence indicate further effects of breathing high oxygen levels in animals and healthy subjects [1, 42], but evidence of pulmonary toxicity in a clinical scenario is limited [43]. The pathological features of this condition are commonly referred to as the Lorrain Smith effect [44] and are characterized by tracheobronchitis, which can be accompanied by pleuritic pain, bronchial irritation, cough, and sore throat. Symptoms may spread from the upper airways into the lungs, where diffuse alveolar damage manifests and contributes to edema, vascular leakage, arteriolar thickening, pulmonary fibrosis, and emphysema, reflected by progressive paradoxical hypoxia, dyspnea, and tachypnea. Additionally, prolonged hyperoxic exposure alters the microbial flora in the upper airways and further increases the risk of secondary infections and lethality. Notably, these pulmonary effects are often in addition to the primary (e.g., pneumonia) and secondary (e.g., ventilator-induced lung injury) lung injury, which are accompanied by inflammatory responses.

The central nervous system is typically the first to suffer from the effects of excessive ROS formation. The spectrum of neurological symptoms is referred to as the Paul Bert effect and ranges from nausea, dizziness, and headache to vision disturbances (retinal damage), neuropathies, paralysis, and convulsions [1].

Vascular effects of hyperoxia have been well documented and may have both harmful and beneficial effects. Arterial hyperoxia increases the systemic vascular resistance and induces vasoconstriction, which may impair organ perfusion, especially in the cerebral and coronary region [45–47]. Accompanying cardiovascular alterations result from even short-term exposure and include a decrease in heart rate, stroke volume, and cardiac output [48]. However, hyperoxia is not a universal vasoconstrictor in all vascular regions, and blood flow may be redistributed to the hepatosplanchnic circulation in septic shock [1, 49]. Alternatively, the administration of oxygen promotes hemodynamic stabilization during vasodilatory shock, decreases intracranial pressure by cerebral vasoconstriction, and preserves tissue oxygenation during hemodilution [2, 50].

### Clinical studies

#### Critical care

Recent studies assessing the clinical effects of arterial hyperoxia or normobaric supplemental oxygen in critical care are listed in Table 1. As highlighted in recent meta-analyses [51, 52], the effects on major clinical endpoints are conflicting and may be partially explained by heterogeneous methodology and subgroup differences in critically ill patients. Pooled effect estimates favoring normoxia are quite consistent, but the harmful effects were previously shown to be impacted by the definition of hyperoxia and may be more pertinent to specific subgroups and at specific moments of admission.

**Table 1:** Studies assessing the clinical effects of arterial hyperoxia or supplemental oxygen in subgroups of critically ill patients

Author	Country	Study type	Inclusion period	Subgroup	Sample size	Harm	Conclusions
Eastwood et al. [57] (2012)	Australia and New Zealand	Cohort	2000–2009	MV	152,680	–	Hypoxia in first 24 h of admission was associated with increased in-hospital mortality, but hyperoxia was not.
de Jonge et al. [56] (2008)	The Netherlands	Cohort	1999–2006	MV	36,307	+	High FiO_2_ and both low PaO_2_ and high PaO_2_ in first 24 h of admission were associated with in-hospital mortality
Suzuki et al. [96] (2014)	Australia	Before-after pilot	2012	MV	105	+/–	Conservative oxygen therapy in mechanically ventilated ICU patients was feasible and free of adverse biochemical, physiological, or clinical outcomes while allowing a marked decrease in excess oxygen exposure
Aboab et al. [41] (2006)	France	Experimental	NA	ARDS	14	+/–	In mechanically ventilated patients with ARDS, the breathing of pure oxygen leads to alveolar derecruitment, which is prevented by high PEEP
Austin et al. [53] (2010)	Australia	RCT	2006–2007	COPD	405	+	Titrated oxygen treatment significantly reduced mortality, hypercapnia, and respiratory acidosis compared with high-flow oxygen in acute exacerbations of COPD
Cameron et al. [55] (2012)	New Zealand	Cohort	2005–2008	COPD	180	+	Serious adverse clinical outcomes are associated with both hypoxaemia and hyperoxaemia during acute exacerbations
Perrin et al. [54] (2011)	New Zealand	RCT	2007–2009	Asthma	106	+	High-concentration oxygen therapy causes a clinically significant increase in transcutaneous CO_2_ during severe exacerbations
Bellomo et al. [63] (2011)	Australia and New Zealand	Cohort	2000–2009	CA	12,108	–	Hyperoxia did not have a robust or consistently reproducible association with mortality
Elmer et al. [62] (2014)	USA	Cohort	2008–2010	CA	184	+	Severe hyperoxia was independently associated with decreased survival to hospital discharge
Ihle et al. [64] (2013)	Australia	Cohort	2007–2011	CA	584	–	Hyperoxia within the first 24 h was not associated with increased hospital mortality
Janz et al. [61] (2012)	USA	Cohort	2007–2012	CA	170	+	Higher levels of the maximum measured PaO_2_ were associated with increased in-hospital mortality and poor neurological status on hospital discharge
Kilgannon et al. [59] (2010)	USA	Cohort	2001–2005	CA	6326	+	Arterial hyperoxia was independently associated with increased in-hospital mortality compared with either hypoxia or normoxia
Kilgannon et al. [60] (2011)	USA	Cohort substudy	2001–2005	CA	4459	+	Supranormal oxygen tension was dose-dependently associated with the risk of in-hospital death
Kuisma et al. [69] (2006)	Finland	RCT pilot	NA	CA	28	–	No indication that 30 % oxygen with SpO_2_ monitoring did worse than the group receiving 100 % oxygen
Lee et al. [65] (2014)	Korea	Cohort	2008–2012	CA	213	–	Mean PaO_2_ was not independently associated with in-hospital mortality
Nelskyla et al. [112] (2013)	Australia	Cohort	2008–2010	CA	122	–	No statistically significant differences in numbers of patients discharged from the hospital and 30-day survival between patients with hyperoxia exposure and no exposure
Spindelboeck et al. [67] (2013)	Austria	Cohort	2003–2010	CA	145	–	Increasing PaO_2_ was associated with a significantly increased rate of hospital admission and not with harmful effects
Vaahersalo et al. [66] (2014)	Finland	Cohort	2010–2011	CA	409	–	Hypercapnia was associated with good 12-month outcome, but harm from hyperoxia exposure was not verified
Minana et al. [113] (2011)	Spain	Cohort	2003–2009	ADHF	588	–	Admission PaO_2_ was not associated with all-cause long-term mortality
Ranchord et al. [114] (2012)	New Zealand	RCT pilot	2007–2009	STEMI	136	–	No evidence of benefit or harm from high-concentration compared with titrated oxygen
Stub et al. [78] (2012)	Australia	RCT	2011–2014	STEMI	441	+	Supplemental oxygen therapy in patients with STEMI but without hypoxia increased myocardial injury, recurrent myocardial infarction, and cardiac arrhythmia and was associated with larger myocardial infarct size at 6 months. Further results anticipated.
Sutton et al. [115] (2014)	Australia and New Zealand	Cohort	2003–2012	Post cardiac surgery	83,060	–	No association between mortality and hyperoxia in the first 24 h in ICU after cardiac surgery
Ukholkina et al. [80] (2005)	Russia	RCT	NA	AMI	137	–	Inhalation of 30–40 % oxygen within 30 min prior to endovascular myocardial reperfusion and within 4 h thereafter reduced the area of necrosis and peri-infarction area, improved central hemodynamics, and decreased the rate of post-operative rhythm disorders as compared with patients breathing ambient air
Zughaft et al. [116] (2013)	Sweden	RCT	NA	ACS	300	–	The use of oxygen during PCI did not demonstrate any analgesic effect and no difference in myocardial injury measured with troponin- t or in the morphine dose
Asher et al. [89] (2013)	USA	Cohort	NA	TBI	193	–	PaO_2_ threshold between 250 and 486 mm Hg during the first 72 h after injury was associated with improved all-cause survival independently of hypocarbia or hypercarbia
Brenner et al. [88] (2012)	USA	Cohort	2002–2007	TBI	1547	+	Hyperoxia within the first 24 h of hospitalization was associated with worse short-term functional outcomes and higher mortality
Davis et al. [87] (2009)	USA	Cohort	1987–2003	TBI	3420	+	Both hypoxemia and extreme hyperoxemia were associated with increased mortality and a decrease in good outcomes
Quintard et al. [83] (2014)	Switzerland	Cohort	2009–2013	TBI	36	+	Incremental normobaric FiO_2_ levels were associated with increased cerebral excitotoxicity independently from brain tissue oxygen and other important cerebral and systemic determinants
Raj et al. [90] (2013)	Finland	Cohort	2003–2012	TBI	1116	–	Hyperoxemia in the first 24 h of admission was not predictive of 6-month mortality
Rincon et al. [92] (2013)	USA	Cohort	2003–2008	TBI	1212	+	Arterial hyperoxia was independently associated with higher in-hospital case fatality
Jeon et al. [84] (2014)	USA	Cohort	1996–2011	Stroke	252	+	Exposure to hyperoxia was associated with delayed cerebral ischemia
Rincon et al. [85] (2014)	USA	Cohort	2003–2008	Stroke	2894	+	Arterial hyperoxia was independently associated with in-hospital death as compared with either normoxia or hypoxia
Ali et al. [82] (2014) and Roffe et al. [117] (2011)	UK	RCT pilot	2004–2008	Stroke	289	–	Routine oxygen supplementation started within 24 h of hospital admission with acute stroke led to a small improvement in neurological recovery at 1 week, but no outcome differences were observed at 6 months
Ronning et al. [81] (1999)	Norway	Quasi-RCT	1994–1995	Stroke	310	+	Supplemental oxygen should not routinely be given to non-hypoxic patients with minor or moderate strokes
Singhal et al. [118] (2005)	USA	RCT pilot	NA	Stroke	16	–	High-flow oxygen therapy is associated with a transient improvement of clinical deficits and MRI abnormalities
Young et al. [91] (2012)	Australia and New Zealand	Cohort	2000–2009	Stroke	2643	–	Worst arterial oxygen tension in the first 24 h was not associated with outcome
Stolmeijer et al. [119] (2014)	The Netherlands	Cohort	NA	Sepsis	83	–	No association between mortality and hyperoxia, nor between lower FiO_2_ and other detrimental effects

NA, not available; +, study found harm from supplemental oxygen or arterial hyperoxia; –, no harm found from supplemental oxygen or arterial hyperoxia

*ACS* Acute coronary syndrome, *ADHF* Acute decompensated heart failure, *AMI* Acute myocardial infarction, *ARDS* Acute respiratory distress syndrome, *CA* Cardiac arrest, *CO*_*2*_ Carbon dioxide, *COPD* Chronic obstructive pulmonary disease, *FiO*_*2*_ Fraction of inspired oxygen, *ICU* Intensive care unit, *MRI* Magnetic resonance imaging, *PaO*_*2*_ Partial pressure of arterial oxygen, *PCI* Percutaneous coronary intervention, *MV* Mechanical ventilation, *PEEP* Positive end-expiratory pressure, *RCT* Randomized control trial, *SpO*_*2*_ Oxyhemoglobin saturation, *STEMI* ST-segment elevation myocardial infarction, *TBI* Traumatic brain injury

It is well established that the use of higher FiO_2_ can lead to progressive hypercapnia during a state of chronic compensated respiratory acidosis, and serious adverse outcomes have been shown in acute exacerbations of chronic obstructive pulmonary disease or asthma [53–55]. Likewise, high fractions of oxygen in the inspired air and arterial blood have been associated with increased mortality in mechanically ventilated patients [56].

Owing to a striking lack of robust clinical trials, a causal relationship is still uncertain and both the magnitude and direction of the associations depend on the adjustment for illness severity scores, FiO_2_, and other confounders [56, 57]. Future randomized controlled studies are urgently needed to definitively elucidate the causal effects of oxygenation targets and derangements on clinical outcomes of critically ill patients.

Excessive oxygenation may be most intensively studied after resuscitation from cardiac arrest as both the vascular alterations and the ischemia and reperfusion injury are hypothesized to be hazardous [58]. In a dose-dependent manner, hyperoxia has been linked to worse outcome in these patients [59–62]. The adverse association was not systematically reproduced and this was possibly due to heterogeneity in study methods [63–68]. The only randomized controlled trial in the post-resuscitation period found that 30 % oxygen ventilation was not worse in comparison with 100 % oxygen, but the study was underpowered to detect significant differences [69]. In view of all recent data, supplemental oxygen administration during resuscitation still appears desirable, but hyperoxia should be avoided in the post-resuscitation phase and saturation should be targeted at 94–96 % [58, 70].

A large number of both experimental and clinical studies have primed pediatricians with great awareness of the risks of hyperoxia. For neonatal resuscitation, the routine use of 100 % oxygen has been abandoned after numerous associations with myocardial, neurological, and kidney injury and retinopathy, inflammation, and increased mortality [71, 72]. However, strict adherence to lower target ranges of oxygen saturation among preterm infants did not significantly reduce disability or deaths [73]. Results from a prospective large-scale meta-analysis investigating the most appropriate level of oxygenation for extremely preterm neonates suggested that functional oxyhemoglobin saturation be targeted at 90–95 % in the post-natal period [74].

Hyperoxia-induced vasoconstriction poses a major concern in the management of acute coronary syndromes, and guidelines increasingly suggest a restriction of supplementary oxygen to only those at increased risk for hypoxia [75]. Indeed, oxygen therapy has not been shown to be beneficial after acute myocardial infarction and may even be harmful, causing a marked reduction in coronary blood flow and myocardial oxygen consumption [76, 77]. The vasoconstriction caused by hyperoxia may be of special concern in the acute setting before reperfusion. The AVOID (Air Verses Oxygen In myocarDial infarction) trial aimed to definitively qualify the role of supplemental oxygen in acute myocardial infarction [78] and found increased myocardial injury, recurrent myocardial infarction, cardiac arrhythmia, and infarct size at 6 months [79]. In contrast, a smaller trial observed a beneficial effect of 30–40 % oxygen inhalation over controls during both occlusion and reperfusion [80]. Hemodynamic effects may also be pertinent to acute ischemic stroke patients, who do not appear to benefit from increased survival after prolonged treatment with oxygen [81, 82].

Despite the theoretical benefit of decreasing intracranial pressure through cerebral vasoconstriction, hyperoxia has repeatedly been associated with delayed cerebral ischemia and increased cerebral excitotoxicity after cerebrovascular incidents [83–85]. Interestingly, the synergistic combination of hyperbaric and normobaric hyperoxia was recently found to have potential therapeutic efficacy in severe traumatic brain injury [86]. However, observational data in patients with traumatic brain injury, ischemic stroke, subarachnoid, or intracerebral hemorrhage remain equivocal [87–92].

#### Perioperative care

Liberal oxygen supply is usually accepted in perioperative care in order to avoid potentially life-threatening consequences of hypoxia during surgery. Further effects of perioperative hyperoxia have been comprehensively summarized in meta-analyses enrolling over 7000 patients and generally showed a reduced risk of surgical site infections and postoperative nausea without luxation of postoperative atelectasis [93, 94]. However, risks may outweigh benefits in specific age groups [39] and different subsets. This was recently highlighted in patients undergoing cancer surgery in whom 80 % oxygen supply in the perioperative setting showed a significantly increased long-term all-cause mortality compared with those randomly assigned to 30 % [95].

### Implications for therapy

Several therapeutic options that limit the harmful effects of hyperoxia can be contemplated, but prevention of excessive oxygenation is likely to be the most effective strategy. A rational approach may be a more conservative administration strategy in which oxygen is titrated to a lower tolerable level in order to prevent iatrogenic harm while preserving adequate tissue oxygenation. Recently, a pilot interventional study showed that conservative oxygen therapy in mechanically ventilated patients in the ICU can be feasible and free of adverse outcomes while decreasing excess oxygen exposure [96]. Importantly, when the risks for severe tissue hypoxia are pronounced, ample oxygen supply remains vital and should be started immediately to increase oxygen delivery and preserve tissue oxygenation. Also, oxygen may aid hemodynamic stabilization and decrease intracranial pressure and can be used to stimulate erythropoietin and increase hemoglobin when intermittent hyperoxia is used as a paradoxical trigger for HIF expression.

Experimental interventions to decrease harm from hyperoxia are targeted at numerous steps in the pathway of ROS-induced damage. The primary source for intervention in the oxidative cycle is inhibition of oxidant generation, either quantitatively or qualitatively. Bleomycin and amiodarone are well-known originators of drug-induced pulmonary disease and should be avoided to minimize preventable ROS formation [97, 98]. Limiting the exposure to other exogenous stimuli or preventing electron leakage in the electron transport chain may protect the mitochondria, but this strategy proves cumbersome in actual practice. Although the clinical applicability has been questioned because of little or no preventative or therapeutic effect, the supply of antioxidant enzymes may be a feasible approach to facilitate the conversion, avoid the intermediate formation, and reduce the concentration of strongly reactive oxidants. However, some of these antioxidants may actually have pro-oxidant properties, depending on their concentration and interaction with other molecules. The neutralizing effect of antioxidants may not be sufficient to secure metabolic stability, even when secondary inflammation is mitigated. Finally, oxidant scavenging can shift the balance toward harm when the role of oxidants in cell signaling pathways is suppressed [99].

As an alternative, pathways of cell integrity, cell death, and inflammation may be targeted to reduce further damage and enhance the defense against oxygen radicals. Experimental research suggests protective effects through modulation of protein kinases [100, 101] and transcription factors [102–105]. Moreover, numerous preclinical studies have demonstrated that manipulation of chemokines, cytokines [13, 106], growth factors [107], receptors [108–110], and DAMPs [11, 12, 111] may limit hyperoxia-induced injury, but these targets all remain to be evaluated at the bedside.

## Conclusions

Although oxygen remains of life-saving importance in critical care, accumulating evidence has demonstrated the prominent role of hyperoxia and the consequent formation of ROS in the pathogenesis of several life-threatening diseases. The toxic effects of supraphysiological oxygen concentrations are driven by cell damage, cell death, and inflammation. These aspects are of special concern in the pulmonary compartment, where absorptive atelectasis impairs respiratory function at high inspiratory oxygen levels. The cerebral and coronary circulations are at specific risk when vascular alterations manifest. Long-term exposure to hyperoxia impairs the innate immune response and increases susceptibility to infectious complications and tissue injury. Given that critically ill patients are prone to inflammation, cardiovascular instability, and depleted antioxidant mechanisms, the most rational practice may be to supply oxygen conservatively and titrate the therapy carefully to the patient’s needs. However, our understanding of oxygen toxicity is limited in humans, and conflicting findings hamper the constitution of compelling guidelines. Further research is warranted to study hyperoxia-induced effects in clinical practice, to elucidate time—and dose-response relationships, and to provide evidence-based oxygenation targets and interventions through robust clinical trials.

## Abbreviations

DAMP: Damage-associated molecular pattern molecule
FiO_2_: Fraction of inspired oxygen
HIF: Hypoxia-inducible factor
ICU: Intensive care unit
PaO_2_: Partial pressure of arterial oxygen
PMN: Polymorphonuclear neutrophil
ROS: Reactive oxygen species
